# Annotations

**Published:** 1909-01-09

**Authors:** 


### Annotations.

### The Paris Student Riots.

The recent excited and disorderly scenes in Paris, in which the medical students of the University played a leading part, are much to be regretted. The cause of the trouble was the introduction of a new certificate for advanced students. There had been several previous "rows" in the student quarter during the past half-year, and the last and most serious disturbance no doubt gained in force and virulence from the preceding fermentation of student feeling and opinion. But as Professor Osler, who was an eye-witness of the last affair, points out in a letter to the *Times*, the chief participants in the riot of December 21 were not medical students in the ordinary sense but students of medicine of an older growth, practitioners of advanced professional standing and attainments. These medical men—candidates for the *agrégé* professorships in the medical sciences in French Schools of Medicine—128 candidates for 20 vacancies—created the disturbance to mark their disapproval of a new regulation which makes the examination in question more preliminary and elementary in character by the inclusion of the subjects of anatomy and physiology. This they regard as an affront, a deliberate reflection upon their knowledge and attainments, and an irksome and provocative waste of time. They deny the necessity for re-examining men of their standing in the elementary subjects. Rumours of a threatened outbreak had been in the air for some days previous to the *concours*, and on December 21 the candidates and other students, amidst scenes of fierce disorder, rushed the examination rooms and forced the adjournment of the examination. At first the police, and later the soldiery, joined in the disturbance, and an element of roughs of the *Apache* type helped to swell the riot and complicate efforts towards order and good feeling. The whole tumultuous and undignified affair was prolonged for many hours: many injuries were received, and a number of arrests were made. The riot formed the text of a rather out-of-date and tactless discursion, in a leading article in the *Times*, upon the innate rowdy tendencies of "the medical man in his larval stage." We expect the halfpenny newspapers to cling to the old conceptions of the medical student as a noisy riotous looter of door-knockers; but things have changed so very greatly in this respect in England and elsewhere since the days of fifty years ago, and educated people know this so well, that semi-facetious remarks upon the manners of English medical students (and incidentally also of "*locum tenentes* so called apparently on the *lucus a non* principle") seem to us to be inappropriate, and not quite in the best of taste. We therefore endorse Dr. Osler's gentle reproof, and express the hope that the usual accuracy and dignified tone of our contemporary in its treatment of medical matters will not again be departed from, even for the sake of a ponderous dig at an almost extinct type of English medical student.

### Kissing the Testament.

We have on previous occasions spoken here of the custom of kissing the Testament in courts of justice, and have expressed our opinion upon this undignified and unhygienic practice. Two recent events, however, tempt us to refer again to the subject. A medical man, apparently ignorant of his right to affirm in the Scottish fashion, lately protested in Court against kissing a dirty Testament. He was handed a cleaner copy and was told that he must kiss that or be committed for contempt of court. It is charitable to suppose that those who thus forced the medical witness to kiss the book were, like him, ignorant of his right to be sworn in the alternative manner. But every medical man and every court official should know by now that it is not necessary for a witness to be Scottish by birth or descent to be excused from the dirty English practice of contaminating his lips with a much-used Testament. The second reason for renewing our protest against kissing the book is afforded by a strongly worded leading article in the *Times* of January 4. This concludes by urging upon Parliament the need for the total abolition of the English practice, "which is degrading in form, disgusting in fact, derogatory to the Bible, dangerous to the people, and altogether alien to the solemnity of an oath and the dignity of a court of justice." A less drastic proposal, which perhaps stands a better chance of success, is that all witnesses should be offered the option of affirming in the Scottish fashion, instead of being practically coerced, as is too often the case, into adopting the English method. The chief reason why court officials almost always try to force witnesses to kiss the book, and place difficulties in the way of affirmation, is that the former practice is the easier and the more expeditious. We can see no other excuse for retaining a custom at once dirty and dangerous. We hope that public opinion may be roused at last, after many ineffectual attempts, to see the matter in its proper light; and that medical men will set a good example by habitually insisting upon being sworn in the dignified Scottish fashion, whereby the perfunctory irreverent mumbling of a subordinate court official is abolished, and the witness, holding up his uncovered right hand, declares with his own voice: "I swear by Almighty God, as I shall answer to God at the great day of judgment, that I will tell the truth, the whole truth, and nothing but the truth." Nothing could be simpler, more impressive, or more binding.

